# Intraosseous Benign Lesions of the Jaws: A Radiographic Study

**DOI:** 10.5812/iranjradiol.7683

**Published:** 2014-01-30

**Authors:** Adineh Javadian Langaroodi, Sima Sadat Lari, Abbas Shokri, Seyed Hossein Hoseini Zarch, Shokofeh Jamshidi, Peyman Akbari

**Affiliations:** 1 Dental Research Center, Department of Oral and Maxillofacial Radiology, School of Dentistry, Mashhad University of Medical Sciences, Mashhad, Iran; 2 Department of Oral and Maxillofacial Radiology, School of Dentistry, Hamedan University of Medical Sciences, Hamedan, Iran; 3 Dental Material Research Center, Department of Oral and Maxillofacial Radiology, School of Dentistry, Mashhad University of Medical Sciences, Mashhad, Iran; 4 Department of Oral and Maxillofacial Pathology , School of Dentistry, Hamedan University of Medical Sciences, Hamedan, Iran; 5 Private Dental Practice, Hamedan, Iran

**Keywords:** Panoramic Radiography, Cystic Lesions, Tumoral Lesions, Maxilla, Mandible

## Abstract

**Background:**

Benign maxillo-mandibular tumors and cysts, which are relatively common findings on radiographs, namely the ubiquitous panoramic view, have to be dealt with by dentists on a daily basis.

**Objectives:**

The aim of this study is to evaluate the panoramic radiographic findings pertaining to benign and tumoral lesions in the maxilla and mandible.

**Patients and Methods:**

Applying a case series method, panoramic images of 61 patients with cysts, benign tumors and tumor-like lesions in the jaws who were referred to Hamedan dental school between 2009 and 2011 were evaluated by two radiologists. They were both blind to histopathological results as well as the objectives of our study. Lesions were assessed based on their location, periphery, internal structure and impaction on the surrounding structures. Then the obtained data were analyzed using descriptive tables.

**Results:**

Cysts were mostly more common in men despite the equal propensity of both genders to benign tumors. In contrast, women showed a higher frequency of tumor-like lesions. The most common site of involvement was the posterior mandible, with peri-apical tooth lesions as the most prevalent dental association. Radiographically, what we most encountered was unilocular radiolucency pertaining to cysts and benign tumors; nevertheless, tumor-like lesions tended to present with a well-defined radiopacity.

**Conclusions:**

Despite its known shortcomings, like every other diagnostic tool, panoramic radiography can contribute to the early detection of maxillary/mandibular lesions that in turn enable the dentist to devise an appropriate treatment plan.

## 1. Background

Given the relatively high incidence of maxillary/mandibular cysts and tumors (1), dentists may have to conduct full radiographic investigations once a preliminary diagnosis is made, in order to determine the extent and characteristics of the lesion that may sometimes be specific enough to make a fairly accurate early diagnosis of a particular tumor (2). In addition, radiographs can occasionally lead to the accidental discovery of lesions with no apparent pertinence to the patient's chief complaint, which is of substantial benefit to both the patient and the physician, as early diagnosis often obviates the need for aggressive therapy (3).

Further studies include intraoral and occlusal radiographs as well as panoramic X-rays, each with their exclusive features. The latter, for instance, provides an overall view of the jaws and teeth structure that cannot normally be achieved by using other visual diagnostic modes. The former, however, fails to capture dento-alveolar lesions (1); whereas, it is well-known for high resolution images owing to direct exposure films, making it ideal for detecting fine details (2). A dentist can be the first health caregiver to come across a variety of lesions; namely cystic, tumoral lesions in the jaws and a deeper insight and better knowledge of radiological clues can contribute enormously to a precise diagnosis. Given the significance of conventional radiography in the diagnosis of maxillary/mandibular lesions, the widespread application of panoramic radiographies in screening procedures and above all, the paucity of studies in this respect, we aimed to evaluate panoramic findings in benign, cystic and tumoral lesions of the jaws in Hamedan.

## 2. Objectives

A dentist can be the first health caregiver to come across a variety of lesions; namely cystic, tumoral lesions in the jaws. Better knowledge of radiological clues can contribute enormously to a precise and due diagnosis. The aim of this study is to evaluate the panoramic radiographic findings pertaining to benign and tumoral lesions in the maxilla and mandible.

## 3. Patients and Methods

Our subjects, 61 cases, were selected among 120 patients with a panoramic radiography as well as histopathological reports pertaining to the lesions detected radiographically. These patients were all referred to Hamedan Dental School with a diverse range of complaints between 2009 and 2011. As it was meant to be a case study, data were all elicited from archived reports and files. The subjects, 34 males and 27 females, radiographically had cyst (s), benign tumor (s) or tumor-like lesion (s), in which each lesion affected the bone (intra-osseous) or the peripheral soft tissue plus an extension to the adjacent bone structure.

Tumor like lesions ranged from reactive, such as giant cell granuloma and aneurysmal bone cysts, to fibro-osseous lesions [cemental dysplasia: periapical and florid cement-osseous dysplasia (PCOD and FLCOD), fibrous dysplasia, cemento-ossifying fibroma (COF) and peripheral ossifying fibroma (POF)]. The X-ray apparatus used also differed from case to case: planmeca Model 2002 cc panoramic machine (planmeca Co., Helsinki, Finland), CR system (cassette system with photostimulable phosphor plates) and Digora PCT (Sordex Co, Helsinki, Finland). Two qualified radiologists reviewed the images separately under uniform light on EIZO MX241W monitor (EIZO NANO Corporation, Japan) and view box for digital and analogue images. Images were also subjected to contrast, zoom and density adjustment if necessary. Observers had no knowledge of the histopathological results as well as the objectives of our study. In case they did not concur, a third opinion was sought through consultation with another expert who was also blind to pathology reports.

Lesions were assessed based on their location, periphery and internal structure. As far as the location was concerned, the lesions could be single-focal, multi-focal and generalized. They could also be situated in the anterior (incisor-canine) and posterior (pre-molar/molar/ramus mandible)/tuberosity (maxilla) of the mouth, or even extend from anterior to posterior. The peripheries of the lesions were described as either well-defined or ill-defined. The former could be corticated, sclerotic and non-corticated (punched-out). Ill-defined borders were either blending or invasive. The third variable, internal structure included three categories: radiolucent, radiopaque and mixed (radiolucent-radiopaque). A range of findings were also studied including root resorption, tooth displacement, cortical perforation, pathologic fracture, widening of periodental ligament (PDL), mandibular canal displacement due to mandibular lesions and maxillary sinus and nasal floor displacement owing to maxillary lesions.

Having the findings assessed, already prepared checklists were filled for every individual case that encompassed clinical, radiographic and histopathologic sections. A third observer carried out the latter. If other clinically pertinent variables such as cortical expansion were necessary, the patients records were evaluated. Descriptive statistical analysis was finally carried out using SPSS software, Ver. 16.1 (SPSS Inc., Chicago, IL, USA).

## 4. Results

In a population of 71 patients (34 males and 37 females) with an average age of 36±12.6 years (6-65), panoramic radiographs showed 31 cysts, 12 benign tumors and 18 tumor-like lesions. Odontogenic keratocyst (OKC) was observed to be the most common diagnosis among cysts, whereas ameloblastoma and giant cell granuloma [central (CGCG) and peripheral (PGCG)] were the most frequent findings in the benign tumor and tumor-like lesion category, respectively (Table 1). With gender as a variable, cysts were more common among women whereas men were affected by tumor-like more than other lesions. Benign tumors, however, were found with an equal frequency in both genders. Benign tumors showed an inclination towards younger age groups in comparison with cysts and tumor-like lesions. Distribution of lesions also varied substantially, with 93% of the lesions being single focal and FLCOD (absolute frequency 4) was the only lesion with multi-focal distribution. In general, the posterior region was the most common site of involvement in all three lesion types. Cysts were also shown to extend from anterior to posterior more than the other two lesions (Table 2). 

**Table 1.** Distribution of Different Lesions

Type of Lesions	Classification of Lesions	Pathologic Diagnosis	Frequency in Every Group No. (%)
**Cysts**	Odontogenic	Odontogenic keratocyst	9 (29)
		Dentigerous cyst	6 (19.5)
		Radicular cyst	6 (19.5)
		Residual cyst	2 (6.5)
		Calcifying odontogenic cyst	2 (6.5)
	Nonodontogenic	Nasopalatin duct cyst	4 (12.9)
	Pseudocyst	Simple bone cyst	2 (6.5)
	Total		31 (100)
**Benign Tumors**	Odontogenic	Ameloblastoma	5 (41.7)
		Odontoma	3 (25)
		Adenomatoid odontogenic tumor	1 (8.3)
		Ameloblastic fibroma	1 (8.3)
	Nonodontogenic	Osteoma	2 (16.7)
	Total		12 (100)
**Tumor-Like Lesions**	Reactive	Central giant cell granuloma	2 (11.1)
		Peripheral giant cell granuloma	4 (22.2)
	Fibro-osseous	Cemento-ossifying fibroma	2 (11.1)
		Peripheral ossifying fibroma	1 (5.6)
		Peri-apical cement-osseous dysplasia	5 (27.8)
		Florid cement-osseous dysplasia	4 (22.2)
	Total		18 (100)

**Table 2.** Distribution of the Single-Focal Lesions Based on the Site of Involvement

Involved Jaw	Cysts No. (%)	Benign Tumors No. (%)	Tumor-Like Lesions No. (%)
**Maxilla**			
Anterior	9 (29)	3 (25)	3 (21.4)
Posterior	2 (6.5)	0	1 (7.1)
Anterior to posterior	3 (9.7)	0	0
**Mandible**			
Anterior	1 (3.2)	1 (8.3)	3 (21.4)
Posterior	14 (45.2)	7 (58.3)	6 (42.9)
Anterior to posterior	2 (6.5)	1 (8.3)	1 (7.1)
**Total**	3 1(100)	12 (100)	14 (100)^a^

^a^ Multi-focal lesions have not been considered (including every 4 cases of florid cement-osseous dysplasia)

Altogether, peri-apical tooth lesions were the most prevalent dental association. Impacted tooth coincided with dentigerous cysts, odontoma (one case) and ameloblastoma (two cases) (Table 3). As to the internal structure and margins of the lesions, cysts and benign tumors had a radiolucent appearance as opposed to tumor-like lesions with a dominant radiopaque presentation. Cysts and benign tumors were well-defined with corticated borders, while tumor-like lesions were equally well-corticated and sclerotic. An invasive border was detected only in a case of COF and PGCG while a blending border was absent in all categories (Table 4). 

**Table 3.** Distribution of the Lesions Based on Association with Tooth

	Cysts No. (%)	Benign Tumors No. (%)	Tumor –Like Lesions No. (%)
**Without tooth association**	5 (16.1)	2 (16.7)	7 (38.9)
**With Tooth association**			
Peri-coronal	6 (19.4)	3 (25.0)	0
Peri-apical	17 (54.8)	4 (33.3)	9 (50.0)
Inter-radicular	3 (9.7)	3 (25.0)	2 (11.1)
**Total**	31 (100.0)	12 (100.0)	18 (100.0)

**Table 4.** Distribution of the Lesions Based on Border and Internal Structure

	Border				Internal Structure		
	Well-Corticated No. (%)	Sclerotic No. (%)	Non-Corticated No. (%)	Invasive No. (%)	Radiolucent No. (%)	Radiopaque No. (%)	Mixed No. (%)
**Cysts**	26 (83.9)	1 (3.2)	4 (12.9)	0	30 (96.8)	0	1 (3.2)
**Benign Tumors**	10 (83.3)	0	2 (16.7)	0	6 (50.0)	5 (41.7)	1 (8.3)
**Tumor-Like Lesions**	6 (33.3)	6 (33.3)	4 (22.2)	2 (11.1)	6 (33.3)	7 (38.8)	5 (27.7)

Bone expansion is normally invisible on panoramic radiographs owing to a technical default obstructing our view to the buccal and lingual plate. Therefore, we decided to investigate it clinically. It was found to be more commonly associated with benign tumors (58%) compared to cysts (51%) and tumor-like lesions (44%). Root resorption was associated with OKC and ameloblastoma in cysts and the benign tumor group, respectively. It was also linked with giant cell granuloma and a case of FLCOD in the tumor-like lesion group (Table 5). 

There was also an association between tooth displacement and benign tumors. However, displacement of the sinus walls, nasal floor and mandibular canal was more common in cystic lesions (Table 6). Cortical perforation was only found in four cases with odontogenic keratocysts. There was no trace of any pathologic fractures in panoramic radiographs. PDL widening was detected only in a case of COF and PGCG.

**Table 5.** Distribution of the Lesions Based on Root Resorption and Tooth Displacement

	External Root Resorption			Tooth Displacement		
	Yes No. (%)	No No. (%)	Total No. (%)	Yes No. (%)	No No. (%)	Total No. (%)^a^
**Cysts **	7 (26.9)	19 (73.1)	26 (100.0)	14 (53.8)	12 (46.2)	26 (100.0)
**Benign Tumors**	4 (36.4)	7 (63.6)	11 (100.0)	8 (72.7)	3 (37.3)	11 (100.0)
**Tumor-Like Lesions**	3 (21.4)	11 (78.6)	14 (100.0)	7 (50.0)	7 (50.0)	14 (100.0)

^a^ The total number of cases in each group that occurred in the tooth bearing area

**Table 6.** Distribution of the Lesions Based on Displacement of Around Structures

Displacement of Around Structures	Cysts No. (%)	Benign Tumors No. (%)	Tumor-Like Lesions No. (%)
**Yes**	18 (66.7)	3 (42.8)	3 (27.2)
**No**	9 (33.3)	4 (57.2)	8 (72.8)
**Total^a^**	27 (100.0)	7 (100.0)	11 (100.0)

^a^ The total number of cases in each group that occurred in close proximity to the surrounding structures (nasal and sinus floor, mandibular canal)

## 5. Discussion

Radiographs are ordered when clinicians, bearing clinical evidence as well as past history of the patient in mind, intend to investigate their case at hand further, or to corroborate their clinical suspicion with regard to the list of differential diagnoses. Panoramic radiographies, in particular, are commonly used to assess dento-alveolar structures. This study investigated panoramic findings in 61 patients whose histopathological reports were beyond doubt or obscurity, indicative of cysts, benign tumors or tumor-like lesions, located either intraosseously or peripherally with bony impaction in the maxilla/mandible area.

Cysts had a predilection for male gender while benign tumors were equally distributed. Hosseini Zarch (4) attested to our findings in regard to the former; nevertheless, he found a higher incidence of benign tumors in women. Alsyfyani (5) claimed that lesions of cemental dysplasia [PCOD, FLCOD] could be found in 83% of cases in women, which also corroborates our findings in this respect (100%). We also detected female dominance in all tumor-like lesions. Odontogenic keratocysts were unanimously identified as the most prevalent cystic lesion (6, 7). Nonetheless, some private practice-based studies reported a predominance of radicular cysts (8-10). As with other investigations (4, 11), ameloblastoma (41%) and giant cell granuloma were the most common benign tumor and tumor-like lesions, respectively. In case of an intraosseous lesion, attention should be paid to radiological characteristics along with other pertinent findings. These include location, internal structure, border and the impact on the surrounding tissue, with the latter two being critically differentiating between benign and malignant lesions (2). Thus, clinicians must be wary of any flawed interpretation, making a hasty diagnosis based on a single finding. The posterior mandible was the most frequently affected region, in concordance with Sanatkhani's findings (6). Hosseini Zarch (4) also reported that 60% of cysts and benign tumors affected the mandibular area and so did cemental displasia lesions (PCOD and FLCOD). However, this differed from what Varinouskas et al. (12) found, a 63% occurrence of odontogenic cysts in the maxilla. Sanatkhani (6) established a close correlation between cysts and peri-coronal lesions; whereas, our findings pointed to periapical lesions (54.8%).

The predominant radiographic feature in cysts and benign tumors was unilocular lucency, as was confirmed by others (4, 6). The most common radiographic finding in tumor-like lesions including cemental dysplasia was a well-defined radio-opacity. This was in agreement with Hosseini Zarch (4) but challenged by Alsufyani (5), who found 72% of cemental displasias at an intermediate stage (a mixed appearance). In agreement with other studies (6, 10), cortical expansion occurred in 51.6% and 44.4% of cysts and tumor-like lesions, respectively. However, the percentage of cortical expansion in relation to cemental dysplasias was significantly higher than those reported by Alsufyani (5). OKC appears differently on radiography as its epithelium has innate growth potential and thus may cause cortical thinning and perforation (2). They were the only lesions we found related to perforation. Close to what Sanatkhani found (6), the coincidence of root resorption and tooth displacement in cystic lesions were estimated to be 26.9% and 54.8%, respectively; whereas the former was detected in 34.6% of benign tumors.

Despite its known shortcomings like every other diagnostic tool, panoramic radiography can contribute to the early detection of maxillary/mandibular lesions that, in turn enable the dentist to devise an appropriate treatment plan.
